# Two-point kinetic determination of uric acid in serum with uricase, catalase and aldehyde dehydrogenase on the KONE CD analyser

**DOI:** 10.1155/S1463924682000108

**Published:** 1982

**Authors:** G. A. Harff, F. Lamie

**Affiliations:** 1 Department of Clinical Chemistry Academic Hospital Free University De Boelelaan 1117 Amsterdam The Netherlands; 2 Hoechst Holland N.V. Behring Diagnostics Sara Burterhartstraat 25 Amsterdam The Netherlands


Journal of Automatic Chemistry, Volume 4, Number 1 (January–March 1982), pages 35–39

### Two-point kinetic determination of uric acid in serum with uricase, catalase and aldehyde dehydrogenase on the KONE CD analyser

**G. A. Harff**

*Department of Clinical Chemistry, Academic Hospital, Free University, De Boelelaan 1117, Amsterdam, The Netherlands,*

**and F. Lamie**

*Hoechst Holland N.V., Behring Diagnostics, Sara Burgerhartstraat 25, Amsterdam, The Netherlands*

### Introduction

Two methods are commonly used for the determination of uric acid—the first is according to Praetorius and Poulsen [1] and the second was devised by Kageyama [2]. Neither method is readily applicable to the KONE CD analyser (manufactured by KONE OY, Finland).

The new aldehyde dehydrogenase (ALDH) method, proposed by Haeckel [3] and improved by Bartl *et al.* [4], overcomes many of the disadvantages in Praetorius's and Kageyama's methods. This paper presents the results of tests on two commerically available reagents for the ALDH method for their applicability to the KONE CD. One of these reagents is produced by Boehringer Mannheim and is formulated according to Bartl. The other is manufactured by Human and is formulated according to the original Haeckel prescription. Bartl's method avoids the disturbing effect of ethanol-converting enzymes by the addition of oxalic acid and pyrazole, and also has a large linear range due to the use of xanthine, which is a competitive inhibitor of uricase. Moss [5] described in detail the enzymatic determination of uric acid by the equilibrium procedure, measurement of two-point kinetics, and measurement of the initial rate of reaction. Moss showed that two-point kinetic measurement is the best method available.

Due to the long lag time of the KONE CD's initial reading, a blank-corrected equilibrium method cannot be used. Normal end-point determination appears to be slow and reagent consuming. It was therefore decided to optimize the two-point kinetic method for use on the KONE CD.

### Materials

### Reagents

*Uric acid, ALDH method, No. 242616 supplied by Boehringer Mannheim, FR Germany.*

Each kit contains one bottle of buffer and ethanol (66 ml); three bottles of NADP, catalase, and ALDH (lyophilized); one bottle of uricase (300 μl). When the lyophilisate is dissolved in 20 ml of buffer, and 15 ml of demineralized water and 100 μl of uricase solution are added, the concentrations of the reactants are:

| | |
|---|---|
| Pyrophosphate buffer: | 45 mmol/l |
| Pyrazole: | 70 mmol/l |
| Sodium oxalate: | 70 mmol/l |
| Xanthine: | 0·24 mmol/l |
| Ethanol: | 1·71 mol/l |
| NADP: | 0·57 mmol/l |
| Catalase: | 570 kU/l |
| ALDH: | 570 U/l |
| Uricase: | 186 U/l |

*Fully enzymatic uric acid, No. H1004 manufactured by Human of Taunusstein, FR Germany.*

Each Human kit contains two bottles of buffer and ethanol (50 ml each); three bottles of NAD, catalase, and ALDH (lyophilized); and one bottle of uricase (600 μl). When the lyophilisate is dissolved in 25 ml of buffer, and 200 μl of uricase solution is added, the concentrations of the reactants are:

| | |
|---|---|
| Pyrophosphate buffer: | 42 mmol/l |
| Ethanol: | 1·43 mol/l |
| $NAD^+$: | 0·706 mmol/l |
| Catalase: | 933 kU/l |
| ALDH: | 333 U/l |
| Uricase: | 30 U/l |

The xanthine was supplied by Fluka A.G. of Buchs, Switzerland, and xanthine oxidase by the Calbiochem-Behring Corporation of La Jolla, USA.

### Specimen

The calibration serum used was Calibrated Automated Lock-in, lot No. 1217049, lyophilized human serum from General Diagnostics of the USA. The control sera were Autonorm bovine lot No. 218 supplied by Nyegaard, bovine serum lot No. 0864058 from General Diagnostics, and bovine serum lot No. K 5444-1 from Wellcome Reagents Ltd. The Human controls used were Ortho normal control serum-unassayed lot No. 11T222 and Ortho abnormal control serum-unassayed lot No. 12T322 from Ortho Diagnostics, Belgium. Sera from patients at the Academic Hospital were used in the correlation studies. The specimens were collected in evacuated blood-collection tubes, and were centrifuged within 15 min. of clotting. Serum was assayed on the reference instrument; the interval of testing between instruments was less than 3 h.

### Apparatus

The two instruments used were the KONE CD (also known as OLLI CD) which is made by KONE OY, SF-0232 Espoo, Finland; and an SMA 6/60 Autoanalyzer, manufactured by the Technicon Instruments Corporation, Tarrytown, NY 10591, USA.

0142-0453/82/0401 0035 $05·00 © 1982 Taylor & Francis Ltd

### Experiments and results

### *KONE CD procedures*

All experiments were carried out at 30°C. The samples were added to the cuvettes with physiologic saline to a volume of 250 $\mu$l, and then 300 $\mu$l of reagent solution was added. The minimum final volume required for the absorbance readings with KONE cuvette block No. 384 is 500 $\mu$l. Each cuvette block contains 24 cuvettes. The wavelength of measurement is 340 nm.

### Boehringer Mannheim kit

To test whether the reaction follows pseudo-first-order kinetics, $-\ln(A_n - A_t)$ is plotted against time (see figure 1 where $A_n$ = absorbance after completion of the reaction, and $A_t$ = the absorbance at time $t$ after starting the reaction). The reagent solution was prepared by dissolving a bottle of lyophilisate in 20 ml of buffer and adding 100 ul of uricase (see curve I in figure 1). To test whether this solution can be diluted further, a 20 ml aliquot was diluted with 14 ml of demineralized water (dilution factor 1·7; see curve II in figure 1) and another 20 ml aliquot was diluted with 28 ml of demineralized water (dilution factor 2·4; see curve III in figure 1). Under all three reaction conditions, the concentration of uric acid in the cuvette is 60 $\mu$mol/l.

As figure 1 illustrates, the reaction does follow pseudo-first-order kinetics over the measurement interval both for diluted and undiluted reagents. Table 1 presents the regression equations from the pseudo-first-order kinetics at various uric acid concentrations (40–120 $\mu$mol/l) with a reagent dilution factor of 1·7. All the regression equations obtained for the different uric acid concentrations are linear. The variation in slope (rate constant) is significant. This variation is similar to the slope obtained with undiluted reagent and with reagent diluted 2·4 times with demineralized water. However, despite this variation in slope at reagent dilution factor 1·7, a good linear uric acid dilution curve is obtained.

*Table 1. Comparison of pseudo-first-order kinetics at five uric acid concentrations [$x$ = time (in s) and $y = -\ln(A_n - A_t)$]. The reagent was diluted 1·7 times with demineralized water.*

| Concentration of uric acid in the cuvette ($\mu$mol) | $y = ax + b$ | $S_a$ | $S_b$ | $S_{yx}$ |
|---|---|---|---|---|
| 40 | $y$=0·02114 $x$+1·573 | 0·00028 | 0·027 | 0·018 |
| 60 | $y$=0·01994 $x$+1·217 | 0·00018 | 0·019 | 0·015 |
| 80 | $y$=0·01706 $x$+1·016 | 0·00007 | 0·007 | 0·008 |
| 100 | $y$=0·01520 $x$+0·855 | 0·00007 | 0·007 | 0·009 |
| 120 | $y$=0·01422 $x$+0·716 | 0·00006 | 0·006 | 0·008 |

### *Linearity*

The linear relationship between the uric acid concentration and $\Delta A_{340nm}$ is shown in figures 2 and 3. The variables are the uricase concentration (figure 2), the time of measurement, and the dilution of the reagent with demineralized water (figure 3). With regard to the uricase concentration in the cuvette, 50·5 U/l of uricase gives linearity up to 60 $\mu$mol/l uric acid in the cuvette and at 101 U/l uricase, the linear range extends at least to 120 $\mu$mol/l. Since the linear range obtained with 101 U/l uricase is quite sufficient, the linearity at higher concentrations of uricase was not investigated.

Dilution of the reagent with demineralized water and the time of the first measurement affect the linearity as shown in figure 3. When the first measurement is taken soon (i.e. 30 s) after starting the reaction, the linearity is dependent on the extent of the dilution. Of the first measurement is taken 50 s after starting the reaction, the three concentrations give similar curves, i.e. small variations in the concentration of reagent in the cuvette have no influence on the $\Delta A$ measured. Moreover, when the first measurement is taken at 50 s the reaction proves to be linear up to an initial uric acid concentration in the cuvette of 120 $\mu$mol/l.

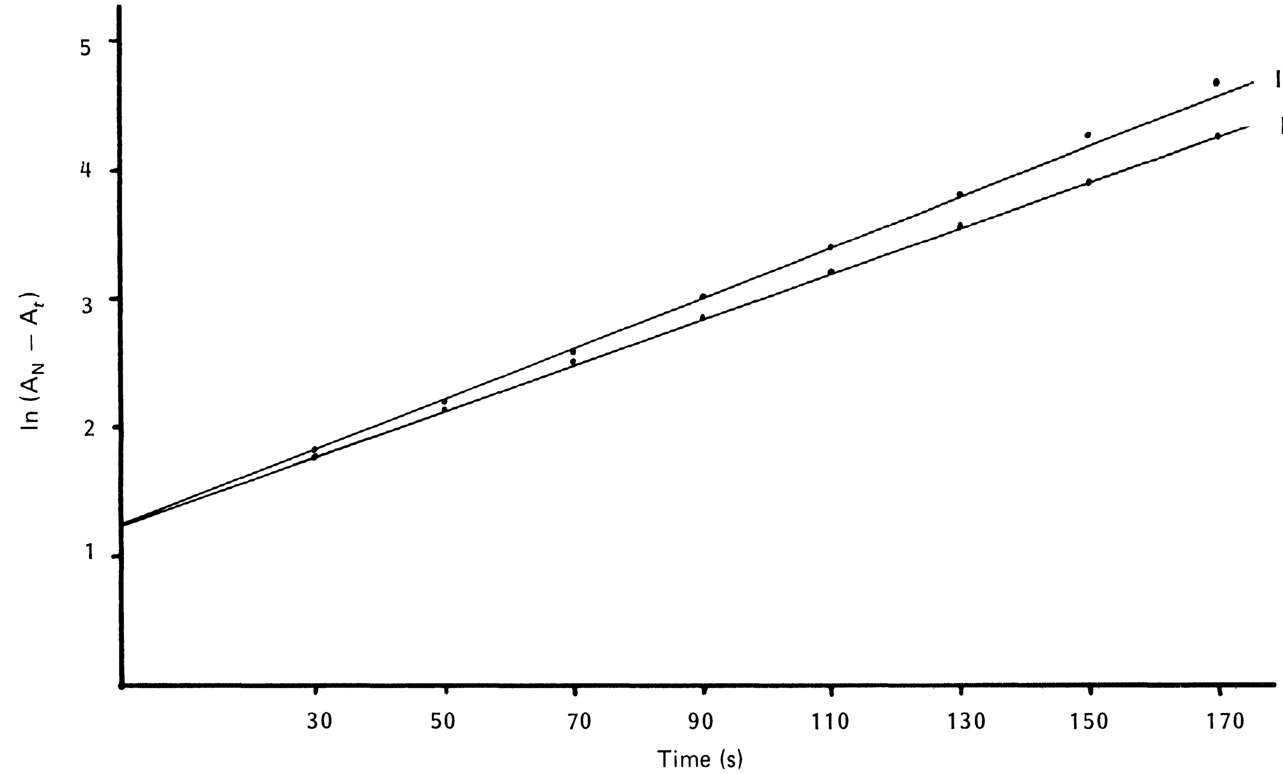

*Figure 1. The effect of diluting the reagent on the reaction with uric acid. Where $A_t$ = absorbance at time t; $A_n$ = absorbance after completion of the reaction. The wavelength of measurement was 340 nm. Initial concentration of uric acid in the cuvette was 60 $\mu$mol/l. Curve I describes the undiluted reagent; curve II the reagent diluted 1·7 times with demineralized water; and curve III is the reagent diluted 2·4 times with demineralized water.*

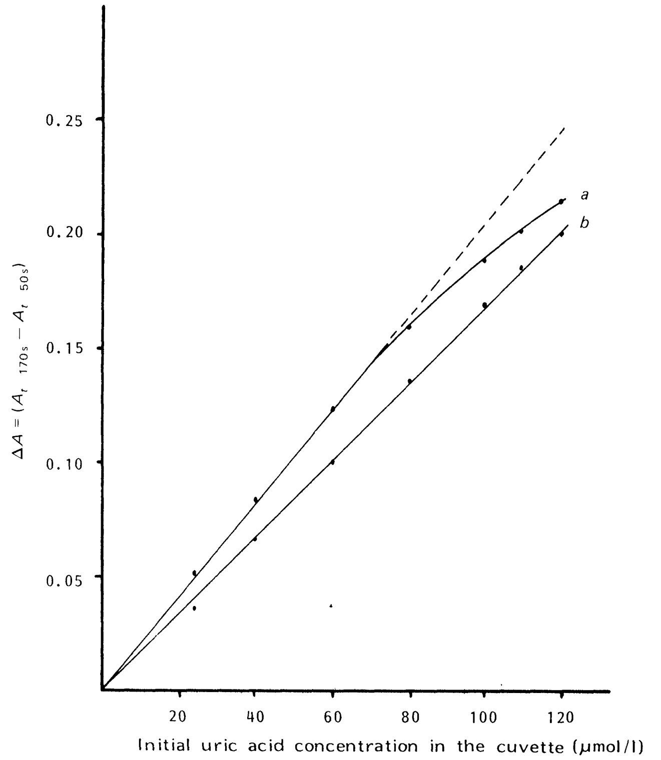

*Figure 2. The effect of uricase concentration on the linearity of the uric acid determination. The reagent was diluted 1·7 times. The concentration of uricase in the cuvette was 50·5 U/l in graph (a) and 101 U/l in graph (b).*

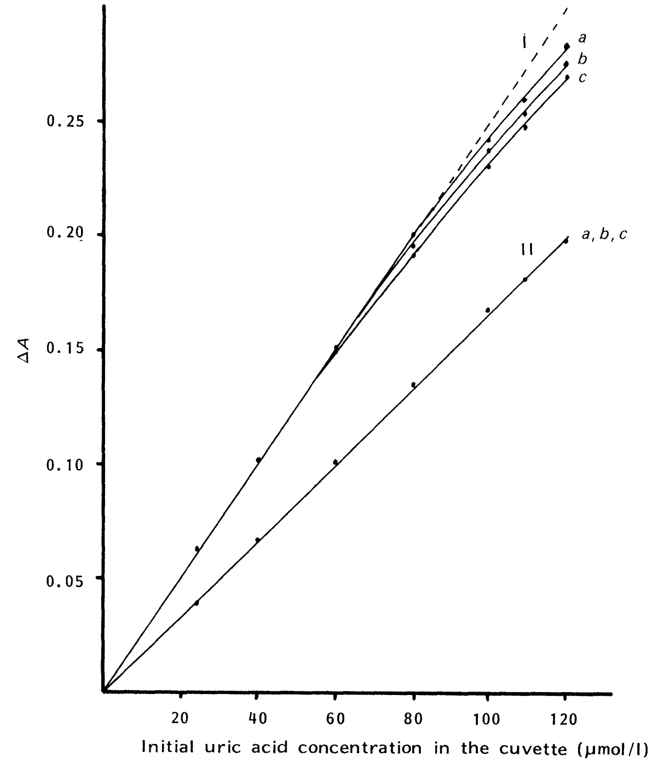

*Figure 3. Effect diluting the reagent and the timing of the absorbance readings on the linearity of the uric acid determination. For graph I* $\Delta A = (A_{t=150s} - A_{t=30s})$*, and for graph II* $\Delta A = (A_{t=170s} - A_{t=50s})$*; graph (a) is undiluted reagent, graph (b) shows the reagent diluted 1·7 times with demineralized water; and graph (c) is the reagent diluted 2·4 times with demineralized water.*

This sensitivity, however, decreases if the first measurement is taken at 50 s.

The KONE CD is connected electronically to the KONE C. As a result, it is possible to determine accurately the time lag between the addition of the reagent and the measurement. The instrument has a minimum lag time between the addition of reagent to three rows of cuvettes in the cuvette block and the first measurement. The shortest possible lag time varies between the several program versions on the tape which is used to program the KONE C. The best lag time is produced by KONE C's program version 3·2, which has a minimum lag time of 40 s at a measuring time of 2 min. The newest program, program 4·0, has a lag time of 50 s at a measuring time of 2 min.

In view of practical constraints, experiments with the Boehringer Mannheim kit will, in future, be run using a lag time of 50 s.

### *Interferences*

### *Interference from xanthine oxidase*

The Boehringer Mannheim kit contains xanthine in order to increase the Michaelis constant; a test was made to discover whether xanthine oxidase interferes in the determination of uric acid. Shamma'a *et al.* [6] investigated the level of the enzyme activity of xanthine oxidase in serum from patients with liver diseases. They found marked increases in xanthine oxidase in the serum from patients with infectious hepatitis. The values given by Shamma'a *et al.* ranged from 1·9 to 1412 International mU/l throughout the course of the disease. Only slight increases of serum xanthine oxidase were found in patients suffering from other diseases of the liver. Several concentrations of xanthine oxidase were investigated. When 1500 ImU/l was added to sera, using routine assay conditions, an apparent increase in uric acid (about 18 μmol/l) was found. Thus only slight increases in uric acid can be expected at high serum xanthine oxidase levels.

### *Interference from xanthine*

Stepatschuk [7] found, at pathologic serum xanthine concentrations, an apparent decrease in urate with a uricase-based method (Uricaquant, cat. No. 124753, supplied by Boehringer Mannheim). His method uses low uricase concentrations during kinetic measurement.

Since Hande *et al.* [8] noted serum xanthine concentrations as high as 148 mg/l after a patient with Burkitt's lymphoma was treated with allopurinol, sera containing added xanthine up to 1 mmol/l were analysed. At 1 mmol/l of xanthine and at a uric acid concentration of 200 μmol/l an increase of 25 μmol/l was found, at at a uric acid concentration of 600 μmol/l an increase of 40 μmol/l in uric acid was found.

### Human kit

The Human kit was also tested to see if the reaction follows pseudo-first-order kinetics. The experiments were carried out with the following reagents: buffer volumes of 12, 15, 18, 25, 52 and 79 ml; uricase concentrations in the cuvette of 5, 10, 20 and 40 U/l; and uric acid concentrations in the cuvette of 12, 20, 30, 40, 50 and 60 μmol.

Pseudo-first-order kinetics was not obtained under any of these conditions.

### *Linearity*

To find out whether the uric acid dilution curve is linear, dilution curves were set up for all of the above reaction conditions. Non-linearity was found at low uric acid concentrations. At higher uric acid concentrations the curve is linear in relation to the dilution. Non-linearity is more extreme the later the first measurement is taken from starting the reaction. At the shortest possible lag time for 24 cuvettes (40 s using KONE C's program 3·2) all the curves displayed poor linearity. When an LKB 8600 reaction-rate analyser was used a linear dilution curve was obtained (lag time was 10 s), although the reaction still did not follow pseudo-first-order kinetics. A drawback of the KONE CD is that it does not allow a short lag time.

### Routine uric acid determination

For routine determination of uric acid with the KONE CD, it was decided to use the Boehringer Mannheim ALDH method. The KONE dispenser is programmed for 30°C, for a 50 μl sample and for 200 μl of physiologic saline. It is unnecessary to incubate the thermostatted cuvette, so 300 μl of starter reagent is added straight away. This starter reagent is prepared by adding 20 ml of buffer, 15 ml of demineralized water, and 100 μl of uricase to a bottle of lyophilized reagent. Since the linear range extends to a concentration of 120 μmol/l in the cuvette (see figure 3), the linear range for the serum is 1350 μmol/l. For serum determination, this linearity is sufficient. The KONE colorimeter was programmed with KONE program version 4·1 (figure 4). Controls used are Ortho normal and Ortho abnormal; these are both human sera and are part of the Ortho Quality Program supplied by Ortho Diagnostics. Two S.D. limits were applied to these sera for quality control of the results obtained with serum samples from patients.

*Figure 4. KONE C program version 4.0.*

### Day-to-day precision

To test day-to-day precision, 30 separate days' lyophilized bovine control sera were reconstructed with low, medium and high concentrations. These three controls were analysed as unknowns between the patients' sera. The precision for Autonorm was 4·3% (mean concentration 170 μmol/l), for General Diagnostics serum it was 3·5% (mean concentration 452 μmol/l) and for Wellcome serum 2·5% (mean concentration 737 μmol/l).

### Correlation

Since the machine used for routine uric acid determination at the Academic Hospital was changed from a Technicon SMA 6/60 to a KONE CD system a correlation study was made between the two instruments. The phosphotungstate reduction method was used for the SMA 6/60. 100 patients' samples were used in the correlation experiment. The regression equation was calculated using Deming's method, which is recommended by Cornbleet and Gochman [9]. Taking $x$ for the SMA 6/60 and $y$ for the KONE CD, the regression equation was $y = 1{\cdot}042\ x - 43{\cdot}1$. $S_{yx} = 15{\cdot}8$ (see figure 5).

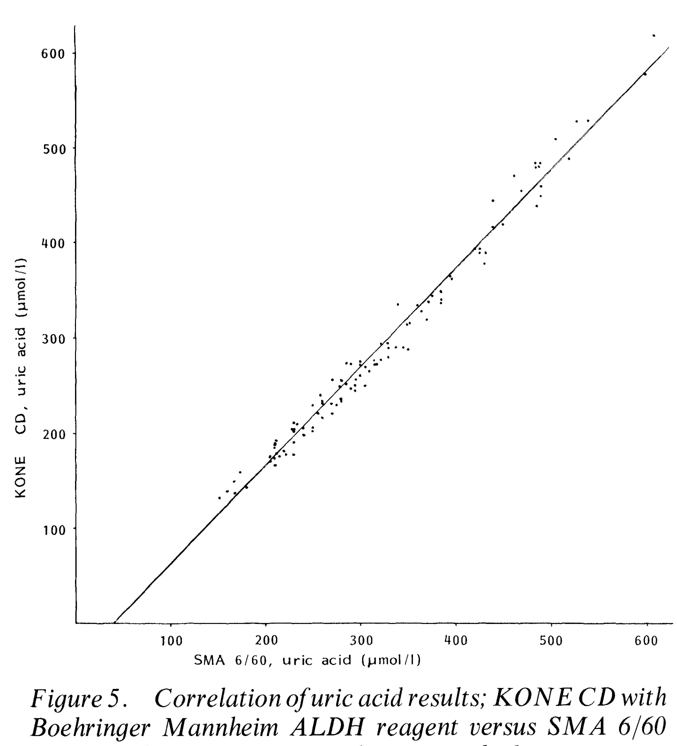

*Figure 5. Correlation of uric acid results; KONE CD with Boehringer Mannheim ALDH reagent versus SMA 6/60 with the phosphotungstate reduction method.*

### Conclusions

The Human H1004 kit cannot be adapted for two-point kinetic determination of uric acid on the KONE CD.

The procedure used with the Boehringer Mannheim kit No. 242616 is quick (allowing 300 determinations/h) and provides good reproducibility of routine use.

In comparison with the phosphotoungstate reduction method, the procedure followed with the Boehringer Mannheim kit gives excellent linearity. Finally, only a minor interference is to be expected in patients' sera from xanthine oxidase and from xanthine.

### Acknowledgements

The authors wish to thank Saskia Bakker and Ellen Bruijnen for their assistance in the preparation of this paper.

### References

1. Praetorius, E. and Poulsen, H., *Scandinavian Journal of Clinical Laboratory Investigations*, **5** (1953), 273.
2. Kageyama, K., *Clinical Chimica Acta*, **31** (1971), 421.
3. Haeckel, R., *Journal of Clinical Chemistry and Clinical Biochemistry*, **14** (1976), 101.
4. Bartl, K., Brandhuber, M. and Ziegenhorn, J., *Clinical Chemistry*, **25** (1979), 619.
5. Moss, D. W., *Clinica Chimica Acta*, **105** (1980), 351.
6. Shamma'a, M. H., Nasrallah, S., Chaglassian, T., Kachadurian, A. K. and Al-Khalidi, U. A. S., *Gastroenterologie*, **48** (1965), 226.
7. Stepatschuk, L., *Clinical Chemistry*, **26** (1980), 789.
8. Hande, K. R., Perini, F., Putterman, G. and Elin, R., *Clinical Chemistry*, **25** (1979), 1492.
9. Cornbleet, P. J. and Gochman, N., *Clinical Chemistry*, **25** (1979), 432.

### Conference announcement

*International Congress on Automation in the Clinical Laboratory*

IUPAC and the Sociedad Española de Quimica Clinica are joint sponsors of this meeting, which will be held in Barcelona, Spain, from 19 to 22 April 1981. The Scientific Committee is chaired by Dr F. L. Mitchell: the provisional scientific programme includes the following:

*19 April*
Opening lecture: The technological revolution in the clinical laboratory, by J. Buttner (FR Germany).

*20 April*
Plenary lecture: Automation in clinical chemistry, current successes and trends for the future, by P. A. Bonini (Italy).

Symposium: Solid phase chemistry, chaired by T. D. Geary (South Africa).

Symposium: Systems for kinetic measurements, chaired by H. L. Purdue (USA).

Symposium: Use of immobilized enzymes, chaired by Ch. Collombel (France).

*21 April*
Plenary lecture: Automation in clinical microbiology, H. Friedman (USA).

Symposium: Instruments for developing countries and for operation in difficult circumstances, chaired by F. L. Mitchell (UK).

Nomenclature for automated analysis (1), by M. Hjelm (UK).

*22 April*
Plenary lecture: Automation in haematology: present and future trends, by S. M. Lewis (UK).

Symposium: Cost analysis, chaired by R. Haeckel (FR Germany).

Nomenclature for automated analysis (2), by M. Hjelm (UK).

Symposium: Computer interfaces and patient identification, chaired by H. Keller (USA).

Symposium: New technology allowing pathology near the patient (clinical chemistry, haematology and microbiology), chaired by A. H. Free (USA).

The congress languages are English, Spanish and French and simultaneous translation will be available during the plenary sessions and symposia. Participants will be able to attend poster sessions, workshops and a commercial exhibition during the three days of the congress.

Papers presented at the congress will be published in *Pure and Applied Chemistry*, abstracts of free communications in this *Journal*.

The registration fee for people over 28 is now 12 000 pesetas, under 28s can register for 6000 pesetas. This does not include travel, hotels or any meals.

*Further details from the Secretariat, International Congress on Automation in the Clinical Laboratory, Apartado de Correos 543, Barcelona, Spain.*

